# Challenges in Participant Engagement and Retention Using Mobile Health Apps: Literature Review

**DOI:** 10.2196/35120

**Published:** 2022-04-26

**Authors:** Saki Amagai, Sarah Pila, Aaron J Kaat, Cindy J Nowinski, Richard C Gershon

**Affiliations:** 1 Northwestern University Feinberg School of Medicine Chicago, IL United States

**Keywords:** mobile phone, mHealth, retention, engagement

## Abstract

**Background:**

Mobile health (mHealth) apps are revolutionizing the way clinicians and researchers monitor and manage the health of their participants. However, many studies using mHealth apps are hampered by substantial participant dropout or attrition, which may impact the representativeness of the sample and the effectiveness of the study. Therefore, it is imperative for researchers to understand what makes participants stay with mHealth apps or studies using mHealth apps.

**Objective:**

This study aimed to review the current peer-reviewed research literature to identify the notable factors and strategies used in adult participant engagement and retention.

**Methods:**

We conducted a systematic search of PubMed, MEDLINE, and PsycINFO databases for mHealth studies that evaluated and assessed issues or strategies to improve the engagement and retention of adults from 2015 to 2020. We followed the PRISMA (Preferred Reporting Items for Systematic Reviews and Meta-Analyses) guidelines. Notable themes were identified and narratively compared among different studies. A binomial regression model was generated to examine the factors affecting retention.

**Results:**

Of the 389 identified studies, 62 (15.9%) were included in this review. Overall, most studies were partially successful in maintaining participant engagement. Factors related to particular elements of the app (eg, feedback, appropriate reminders, and in-app support from peers or coaches) and research strategies (eg, compensation and niche samples) that promote retention were identified. Factors that obstructed retention were also identified (eg, lack of support features, technical difficulties, and usefulness of the app). The regression model results showed that a participant is more likely to drop out than to be retained.

**Conclusions:**

Retaining participants is an omnipresent challenge in mHealth studies. The insights from this review can help inform future studies about the factors and strategies to improve participant retention.

## Introduction

### Background

Today, 85% of the US population owns a smartphone device and daily use averages 4.5 hours [1]. With the rise in smartphone ownership and use, smartphones have become one of the most accessible and cost-effective platforms in health care and research. Smartphones are also pervasive across age, race, and socioeconomic status, allowing researchers to inexpensively reach out to myriad of population-level samples with ease. Specifically, the adoption of mobile health (mHealth) apps—mobile apps that help monitor and manage health of participants through smartphone devices, tablets, and other wireless network devices—has been increasing in the research sphere. The mHealth market is expected to grow at a compound annual growth rate of 17.6% from 2021 to 2028 [2]. In addition, the recent COVID-19 pandemic has led to a rise in the downloads and use of various mHealth apps, highlighting the importance of technology-based remote monitoring and diagnosis for continued advancement in modern health care (eg, [3]).

The greatest advantage of mHealth apps is their convenience. Unlike traditional in-person study settings, mHealth apps can be easily accessed from anywhere at the participant’s convenience. Using apps for remote assessment allows participants to make fewer site visits, substantially reducing the burden of travel and the time needed to participate in laboratory studies. With lowered barriers, it becomes easier for participants to conduct repeated testing and share real-time data based on their daily life experiences. Some mHealth research apps also allow participants to directly communicate with their providers via the app, which may enhance both the effectiveness of the app in its goals (eg, in disease management) and adherence in research studies. Given the ubiquity of smartphones among US adults, mHealth apps for research stand to better meet participants where they are at.

For researchers, the convenience of mHealth apps allows them to reach out to large and diverse participant populations more inexpensively and efficiently than traditional in-person studies. Recently, several large-scale studies were able to recruit thousands of participants within a span of a few months using Apple’s ResearchKit framework (eg, [4-7]). Using these apps, researchers can monitor day-to-day fluctuations of a wide range of real-time data. For example, self-reported emotional outcomes can be assessed together with passive location data to then infer many other real-time variables, such as physical activity, weather, and air quality, that could potentially affect mood throughout the day.

Despite these overwhelming advantages, many mHealth studies experience high participant attrition rooted in the fundamental challenges of keeping participants engaged. For example, consistent with other large-scale mHealth studies, the notable Stanford-led MyHeart Counts study experienced substantial dropout rates; mean engagement with the app was only 4.1 days [8]. It is a ubiquitous problem across all app uses; approximately 71% of app users are estimated to disengage within 90 days of a new activity [9].

It is imperative for mHealth studies to minimize participant dropout, as substantial attrition may reduce study power and threaten the representativeness of the sample. A potential benefit of mHealth research studies should include easier access to well-balanced, representative samples in terms of race, ethnicity, gender, age, education status, etc. However, given that many studies systematically lose participants, systematic differences between participants who are not completing the studies and those who complete the studies, may introduce bias to the sample. Differential retention makes it difficult to conclude whether any observed effects were caused by the intervention itself, retention bias, or inherent differences between groups. Participant dropout also precludes the conduct of longitudinal research.

In an effort to understand the various factors affecting participant retention, recent studies have evaluated recruitment and retention in several remotely conducted mHealth studies. In their cross-study evaluation of 100,000 participants, Pratap et al [10] analyzed individual-level study app use data from 8 studies that accumulated nearly 3.5 million remote health evaluations. Their study identified 4 factors that were significantly associated with increased participant retention: clinician referral, compensation, having the clinical condition of interest, and older age. However, the study only focused on large-scale observational studies led by the Sage or Research Kit, with especially low barriers to entry and exit, thus questioning the appropriateness of applying these findings to other small-scale studies with varying levels of participation. To our knowledge, other published systematic reviews and meta-analyses on engagement and retention are narrowly focused on one subfield of mHealth research, such as depression or smoking, or are only based on a few studies. Thus, it is impossible to extrapolate their findings to other mHealth apps that are not in the same subfield [11-13].

Retention strategies could be incorporated as app features to prevent participant dropout. For example, gamifying mHealth apps by incorporating badges, competitions, and rankings should make the experience more enjoyable and provide better incentives for participants. The addition of reminders, such as push notifications and SMS text messages, and enabling communication with clinicians are also expected to increase participant retention. However, the extent of their effectiveness in successfully engaging and retaining participants is not yet well defined.

### This Study

One fundamental challenge for many mHealth app studies is the rapid and substantial participant dropout. This study aimed to better understand how mHealth studies conducted in the past 5 years have addressed the challenges of participant engagement and retention. We conducted a systematic review of the literature to identify notable factors and strategies used in participant engagement and retention. We hypothesize that participant attrition will be high overall and that there will be shared challenges across different studies that researchers should be cognizant of in future research.

## Methods

### Search Criteria and Eligibility

**Figure 1:**
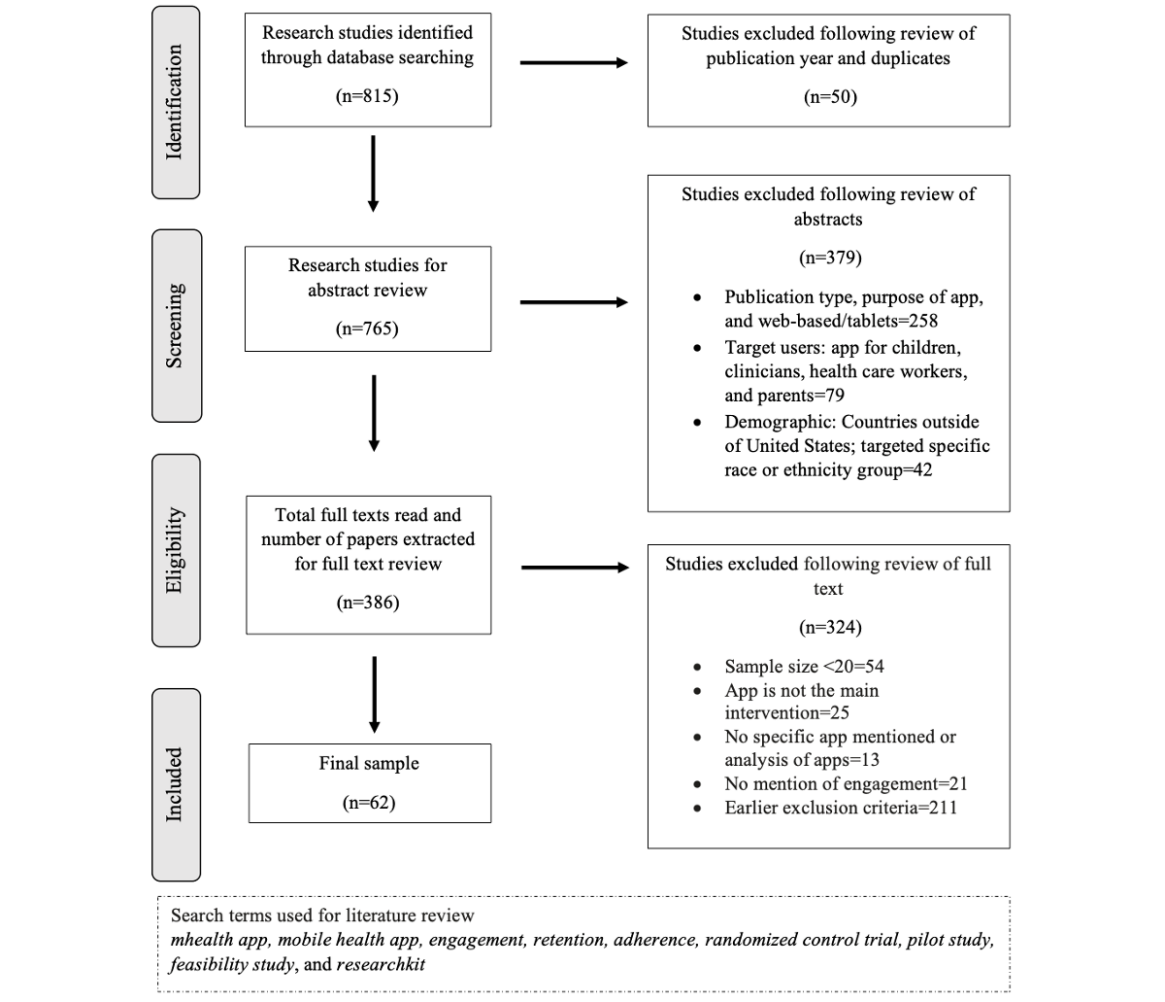
Study selection flowchart.

Our methodology was guided by the PRISMA (Preferred Reporting Items for Systematic Reviews and Meta-Analyses) statement [14]. We identified 3 main databases for this search: PubMed, MEDLINE, and PsycInfo. This review aimed to evaluate the engagement and retention of adults in evaluation research on mHealth apps. Study inclusion criteria were peer-reviewed publications within the last 5 years (January 2015 to October 2020), conducted within the United States, with a minimum of 20 adults. Refer to Figure 1 for more details on the search strategy and exact search terms. Although mHealth takes many forms, we were exclusively interested in mobile-based apps rather than SMS text messaging, tablets, or web-based interventions. We used a variety of research methods and designs, including qualitative, quantitative, or mixed methods. To conduct a comprehensive analysis, we also included mHealth apps in various research areas, ranging from smoking cessation to cardiovascular health research. Articles that were written purely as study protocols or design pieces were excluded. As we were primarily interested in mHealth for intervention purposes, we excluded studies that used fitness app data exclusively (eg, Fitbit and digital pedometers), unless they were specifically geared toward a particular health population (eg, breast cancer survivors and patients with other chronic illnesses). We also excluded evaluations of mHealth apps that focused solely on participant education or where the clinician was the focus of the intervention.

### Data Extraction and Analysis

We initially extracted basic information from each study: title, year, author, target population, operating system, definition of engagement, sample size and type (clinical vs nonclinical), participant age, study duration, main findings, possible implications, and whether participants were compensated. Most of these data were analyzed in a quantitative manner and are described as descriptive statistics in the Results section (ie, app system, sample size, sample type, compensation, and participant age). These data were also used to develop a binomial regression model to determine the factors affecting retention. For the remaining variables, such as the definition of engagement, findings, and implications, we extracted whole sentences or paragraphs that mentioned these items. Following the narrative approach described by Mays et al [15], the first (SA) and second author (SP) analyzed the findings and implications of the initial sample extraction to determine potential themes around retention and engagement. At this point, codes were applied to individual considerations of retention and engagement (or lack thereof) within the articles. After several readings of all extracted findings and implications, the second author initially determined approximately 5 themes related to support and barriers to engagement. These themes were developed from sets of codes, and these sets of codes were considered a *theme* once they were identified in 2 unique articles. After discussion and agreement with the first author, the second author reread the full-text articles to continue to refine these themes and consolidate the findings. We reached saturation when we could no longer identify new themes during the analysis, a process Saunders et al [16] considered *inductive thematic saturation*. Descriptions of these themes are presented in the qualitative findings of the Results section. The definitions of engagement themes and success rates were also processed in a similar way, and they are described in the quantitative findings of the Results section.

## Results

### Final Sample of Studies

After locating all studies that met our search criteria (N=389) and downloading the full text, the first and second authors briefly reviewed the abstracts and full text to determine whether the selected studies met our inclusion criteria. In this process, we confirmed that all the studies that should have been excluded were, in fact, excluded. In a random sample of 100 articles, the authors agreed on 91% (91/100) of these decisions. After reaching an agreement about the remaining 9% (9/100), the first and second author divided the remaining articles for a more detailed review. The final sample comprised 62 articles. Refer to Figure 1 for the study selection flowchart and Multimedia Appendix 1 [4-8,17-73] for characteristics of the studies.

### Descriptive Findings

The mean age across the users of mHealth apps among the 62 studies was 44.14 years (range 32-64.9 years), and the majority were of a clinical population (48/62, 77%). The sample size ranged from 20 (our predetermined minimum) to 101,108 participants, with most studies reporting a sample size of <100 (34/62, 55%). Most studies reported compensating participants (34/62, 55%). Most articles described apps that were available for both iPhone and Android users (29/62, 47%). Refer to Table 1 for more information about the descriptive statistics.

**Table 1:** Descriptive statistics of the 62 studies.

		Values
Age of users (years), mean (range)		44.14^a^ (32-64.9)
**Sample size^b^ (n=108), n (%)**		
	20-49	17 (27)
	50-99	17 (27)
	100-499	12 (19)
	>500	16 (25)
**Platform, n (%)**		
	Android	11 (17)
	iPhone	11 (17)
	Both	29 (46)
	Not reported	11 (17)
**Clinical vs nonclinical, n (%)**		
	Clinical	48 (77)
	Nonclinical	14 (22)
**Compensation, n (%)**		
	Provided compensation	34 (54)
	No compensation	28 (45)
**Success code, n (%)**		
	Not successful	13 (21)
	Partially successful	42 (67)
	Successful	6 (9)
	Not able to calculate	1 (1)

^a^Adequate information to calculate the mean age was not provided for 13 out of the 62 studies. We excluded these studies from the mean age calculation.

^b^The sample size ranged from 20 to 101. The median was 90.5 (IQR 436).

### Definitions

#### Engagement, Retention, Adherence, Compliance, Completion, etc

We identified 2 main themes regarding the use of definitions in the literature sample. Our initial finding was that there is no clear agreement on the definition of engagement. This was likely because the literature in this space varies widely across questions, motivations, and perspectives. The second finding was that engagement was often captured by many different terms. In our sample, we saw terms such as *retention, adherence, compliance, completion*, and others sometimes used interchangeably. Despite this lack of clarity, we categorized our final sample into 3 distinct areas of engagement. Almost all studies (59/62, 95%) described or measured some form of engagement around the opening or using a specific app. Depending on the interface of the app being studied, this open or use definition encompasses nearly any type of app interaction. In some cases, the number of app opens and duration of time spent were collected via a backend system, whereas for others, the data that users logged within the app were part of this definition. The 3 articles that did not fit our open or use category relied on self-reported use of the app or measuring the completion of intervention activities from the app.

#### Success

We asked about the extent to which the research was successful in maintaining participant engagement. Regardless of the term used for engagement or retention, we defined success as the percentage of participants with complete data from the initial sample after an intervention. We defined a *Success Code* variable with 3 categories based on information from the mean and SD. Percentages below the mean minus one SD were considered *not successful* and percentages above the mean plus one SD were considered *successful*. Everything else in between was considered *partially successful* (42/62, 68%). Only 19% (12/62) were considered not successful and 3% (2/62) could not be calculated because they relied on self-reported app use.

Simultaneously, we developed a binomial regression model to examine the factors that could affect retention. The outcome of our binomial regression model was the proportion of complete data from the final sample. The model was weighted on the sample size of the studies. Table 2 shows the odds ratio estimates and CIs from the binomial regression model. For any given participant, it is more likely that they will not be retained than they will be retained. Furthermore, participants with a clinical condition of interest were 4 times more likely to stay in the study than those who did not. Moreover, participants who were compensated were 10 times more likely to stay in the study than those who were not compensated.

**Table 2:** Results from the binomial regression model.

	Odds ratio (95% CI)
Intercept	0.09^a^ (0.093-0.094)
Clinical	4.34^a^ (4.16-4.52)
Compensation	10.32^a^ (9.48-11.25)

^a^*P*<.001.

### Qualitative Findings

Our qualitative findings represent the recurring themes around engagement listed in the findings, discussion, limitations, or conclusions sections of the articles. To be considered a stand-alone theme, the concept must have appeared in at least two independent studies in our sample.

#### Support Themes

We identified 3 major themes (ie, app affordances, successful recruitment, and low barriers to entry) that researchers mentioned that might have kept the participants engaged in their mHealth apps. Even if the article in the sample did not specifically use these supports, we noted where researchers recommended more work to address these supports in future research.

#### App Affordances

Affordances are “the quality or property of an object that defines its possible uses or makes clear how it can or should be used” [74]. In the technology space, this word is often used to describe the possibilities of specific actions that software or hardware allows. On the level of the app being studied (either compared with business-as-usual, another app, or something else altogether), there were several affordances that made research participants more engaged or more likely to stay engaged across the study span. One such factor was gamification. According to Fernandez et al [57], “Gamification or the use of game design elements (badges, leaderboards, rewards, and avatars) can help maintain user engagement.” Very few studies have actually implemented gamification, but this theme was often mentioned as a possibility for future research to evaluate. Approximately one-quarter of our sample mentioned gamification as a future tool for promoting or sustaining engagement in a given mHealth app.

Although it was sometimes an area of interest in its own right, most articles mentioned some level of app reminders, feedback, or notifications that promoted engagement. Indeed, Bidargaddi et al [22] tested the effect of timing on weekends versus weekdays and found that users were most likely to engage with the app within 24 hours if prompted midday on the weekend. It is clear that reminders or other feedback through notifications was a supportive element for producing more engagement and less retention.

Approximately half of the articles mentioned some form of social support provided by coaches or peers within the app. Apps that included a coaching element, either from paraprofessionals, other participants, or the research study team, reported that this social support was critical for maintaining engagement throughout. One specific study by Mao et al [64] reported that 90% of participants who downloaded the app completed 4 months of coaching. This finding was likely because of a combination of participant-selected professional coaches who provided accountability and the social nature of the coaching relationship. In addition to social support, apps featuring tailored and personalized content were more likely to support engagement and adherence to the study.

#### Successful Recruitment

A total of 2 subthemes were drawn from the discussion of recruitment as support for engagement: recruiting highly motivated niche groups and providing some type of motivator in the form of either an incentive or a compensation. mHealth apps that were focused on a niche or highly motivated group of users tended to be more successful in engaging participants over the course of the study. For example, mHealth apps created to support smoking cessation for adult smokers were more likely to be successful when participants were already highly motivated to stop smoking (eg, [18,40,72]). Most studies also mentioned either some form of compensation or other incentives or motivators that could engage more study participants for a longer period. More than half of the studies mentioned providing some type of compensation. Several articles mentioned that there was also a necessary balance needed to use compensation effectively. Providing *too little* incentive might make participants less compelled to continue in the research, but at the same time, providing *too much* incentive could also backfire by reducing their intrinsic motivation to continue. This balance continues to be important for researchers to consider moving forward.

#### Low Barriers to Entry

Related to both app affordances and recruitment strategies, another subtheme that emerged was apps with extremely low barriers to entry. This theme was best described by McConnell et al [7] in the MyHeart Counts Study. Their app was based on Apple’s ResearchKit and enabled nearly 50,000 participants to register and provide consent for research. By launching a free app on smartphones, the authors stated “...the bar for entry to this study was much lower than that for equivalent studies performed using in-person visits. This lowering has the demonstrated advantage that many people consented...” Several other large-scale studies developed using ResearchKit had the advantage of recruiting and enrolling several thousands of participants [75]. This initial engagement was noted as a benefit, but as we learned later, such a low barrier to entry also often meant a low barrier to exit.

#### Barrier Themes

Researchers have also mentioned barriers that might diminish participant engagement. Here, we also noted barriers that were addressed in the discussion or limitations section of the articles, even though they were not actively described in the measures or results. These themes were described as (1) the lack of support codes; (2) low barriers to exit; (3) technical difficulties in using the app; and (4) somewhat counterintuitively, the usefulness of an app.

#### Lack of Support Features

Most barriers, either explicitly described or implied, were those that counteracted the support features. Articles routinely mentioned the lack of app affordances and recruitment success. Research involving apps without gamification, notifications of some sort, or support from peers or coaches was more likely to mention these as potential rationales for poor engagement and areas that could be improved in the future. A similar phenomenon was found in terms of recruitment strategies, where lack of compensation or having a niche group for the app were regularly noted as barriers to retention.

#### Low Barriers to Exit

In the same manner that large smartphone-based studies using the ResearchKit format provided a low barrier to entry, they also provided an equally low barrier to exit. For example, the MyHeart Counts Study further noted that when there is a low barrier to entry, there is a “notable disadvantage that those individuals are by definition less invested in the study and thus less likely to complete all portions” [7]. Almost all the apps available from ResearchKit in our sample represent the highest end of the sample size; however, none of the studies received even a partially successful code in our analysis.

#### Technical Difficulties

Articles that mentioned occasional glitches or *bugs* in the use of their apps were also likely to describe technical difficulties as a reason for lack of engagement. One study explicitly mentioned the use of the research support team to troubleshoot any technical difficulties for users [35], but most articles did not mention how they handled technology support requests. It is likely that some of the technical difficulties could have been on the app side, especially when the apps tested were in a pilot or beta form, but it is also possible that the participants had their own technical difficulties. None of the studies we evaluated performed any kind of pretest to measure participant comfort or familiarity with apps in general or apps similar to the one being studied. Generally, participants who were young adults or middle-aged were assumed to be good with technology overall. In addition, despite nearly a third of the articles mentioning usability and feasibility as a main investigation, only 5 studies mentioned participant results from the System Usability Scale [76], a standardized measure of usability frequently included in the human-computer interaction research space. Otherwise, usability and feasibility analyses were conducted on a study-by-study basis.

#### Usefulness of App

Although it may seem counterintuitive, apps that were extremely useful for participants were also some of the apps anecdotally deemed poor at engagement. For example, participants who successfully quit smoking while using a smoking cessation app generally had poor engagement in the long term. Indeed, if an app *works*, or achieves what it is meant to achieve, and does not offer some kind of regular check-in or maintenance program, it may be reasonable that participants taper the use of the app. In these cases, reduced engagement is a sign of success rather than failure and could actually be considered the goal of the app.

## Discussion

### Principal Findings

This study synthesizes the literature on mHealth apps and the engagement strategies. As mHealth apps continue to grow in popularity and research in this space follows that trend, researchers need to identify what made participants *stay* engaged in the app or studies with the app.

Our review found that most (48/62, 77%) studies were at least partially successful in maintaining participant engagement throughout. Many of these successes were because of the support features of the research or app and the lack of barriers to entry. We determined the categories of strategies that support or detract from engagement. We identified particular elements of the app (eg, feedback, appropriate reminders, and in-app support from peers or coaches) and strategies for research that promote retention (eg, compensation and niche samples) as well as those that do not support retention (eg, lack of support features, technical difficulties, and usefulness of app). Research on the massive population-level ResearchKit apps appeared in both cases, using both successful and unsuccessful engagement and retention techniques. Although low barriers to initial entry could allow thousands of participants to be recruited, the same features also functioned as low barriers to exit. Recruiting a large number of participants is certainly beneficial, but that benefit may be substantially reduced if retention is poor. Future research should consider how to better balance these needs and incorporate factors such as clinical status, referral from providers, and compensation into recruitment plans for population-level apps.

This study used a binomial regression model to assess whether having a clinical condition of interest or receiving compensation affects retention rates. The empirical outcomes of the binomial regression model revealed that (1) any participant is more likely to not be retained than to be retained, (2) participants who have the same clinical condition targeted by the study are 4.33 times more likely to stay in the study than participants who do not have the same clinical condition targeted by the study, and (3) participants receiving compensation are 10.32 times more likely to stay in the study than participants who do not receive compensation. These findings, in line with previous research [10], demonstrate that retaining participants is a true challenge for studies using mHealth apps. Unlike that study [10], we were unable to incorporate clinician referral and age as part of our model because of inconsistent reporting in the articles. Although we planned to include other factors of interest, such as participant gender, income level, years of education, and smartphone platform type, the inconsistent reporting across studies made it challenging to accurately compare these variables. We also recognize that our definition of *success* relies on a normal distribution rather than some other indicator, which might be more appropriate for research with mobile apps that are still in their infancy. To summarize, scientists and researchers must consider different strategies to incentivize and encourage participant retention.

Of course, there is a balance when it comes to successful recruitment strategies, specifically compensation and niche groups. Strong participant engagement or retention may not accurately demonstrate the effectiveness of an app if the participants are overly compensated. Likewise, recruiting a niche group that is highly motivated to use a particular app presents a selection bias and leads to a lack of generalizability of the evaluation findings. Researchers and industry alike would do well to consider this balance when implementing studies using mHealth apps.

### Limitations

Although we offer new insights into mHealth apps and participant engagement, this study has some limitations. First, as a systematic review, we were unable to make claims about all studies on apps. Owing to the file drawer phenomenon and our use of only peer-reviewed published articles, we do not report any studies that might have found null results, even though they might have described *different* interesting supports and barriers for engagement. Therefore, we encourage readers to refrain from generalizations about research on all mHealth apps. Second, we initially extracted information about the diversity of the sample; however, not all articles were clear about the diversity and the possible limitations of their own samples. Unfortunately, we were unable to describe these features in detail, as it is a critical area for more scholarship. Future research should consider the diversity in the demographics of published articles on mHealth apps and provide guidance about that.

### Implications

We recommend that future mHealth apps consider potential support and barriers to participant engagement. Although the promise of moving health experiences onto the devices that people are currently using is great, many of the same barriers to participant engagement still exist and should be considered before moving research onto smartphone administration exclusively.

### Conclusions

Retaining participants is a ubiquitous challenge for studies using mHealth apps. Despite the continued success of mHealth apps in the research sphere, there are many barriers to participant retention and long-term engagement. The insights from this review will help inform future studies about the potential different strategies and factors to consider and improve mHealth app engagement and retention.

## Appendix

Multimedia Appendix 1 Study characteristics of the 62 articles.

## Abbreviations

mHealth: mobile health
PRISMA: Preferred Reporting Items for Systematic Reviews and Meta-Analyses
