# Supplementary material for: Challenges in Participant Engagement and Retention Using Mobile Health Apps: Literature Review
Source: J Med Internet Res. 2022 Apr 26;24(4):e35120. doi: 10.2196/35120 (PMC9092233; doi:10.2196/35120)
Supplement: Multimedia Appendix 1 [file jmir_v24i4e35120_app1.docx]

Multimedia Appendix 1: Study characteristics of the 62 articles

| Study title | Author, year, reference number | Sample size | Study duration (weeks) | Platform | Compensation (yes/no) | Retention (%) |
| --- | --- | --- | --- | --- | --- | --- |
| Engagement and Usability of a Cognitive Behavioral Therapy Mobile App Compared With Web-Based Cognitive Behavioral Therapy Among College Students: Randomized Heuristic Trial | Purkayastha et al, 2020 [17] | 30 | 2 | iPhone and Android | No | 100 |
| The Asthma Mobile Health Study, Smartphone Data Collected Using ResearchKit | Chan et al, 2018 [4] | 5875 | 84 | iPhone | No | 13 |
| Initial Assessment of a Comprehensive Digital Smoking Cessation Program That Incorporates a Mobile App, Breath Sensor, and Coaching: Cohort Study | Marler et al, 2019 [18] | 319 | 16.5 | iPhone and Android | Yes | 85 |
| A Comprehensive Digital Program for Smoking Cessation: Assessing Feasibility in a Single-Group Cohort Study | Patrick et al, 2018 [19] | 41 | 1.3 | iPhone and Android | Yes | 85 |
| Efficacy of the Mindfulness Meditation Mobile App “Calm” to Reduce Stress Among College Students: Randomized Controlled Trial | Huberty et al, 2019 [20] | 88 | 8 | Not reported | Yes | 66 |
| Incorporating Behavioral Trigger Messages Into a Mobile Health App for Chronic Disease Management: Randomized Clinical Feasibility Trial in Diabetes | Sittig et al, 2020 [21] | 20 | 8 | Not reported | No | 60 |
| To Prompt or Not to Prompt? A Microrandomized Trial of Time-Varying Push Notifications to Increase Proximal Engagement With a Mobile Health App | Bidargaddi et al, 2018 [22] | 1225 | 12.7 | Not reported | No | 38 |
| An mHealth App for Self-Management of Chronic Lower Back Pain (Limbr): Pilot Study | Selter et al, 2018 [23] | 93 | 12 | iPhone and Android | No | 38 |
| Pilot Randomized Trial of a Self-Help Behavioral Activation Mobile App for Utilization in Primary Care | Dahne et al, 2019 [24] | 52 | 8 | iPhone and Android | Yes | 87 |
| Physical Activity, Sleep and Cardiovascular Health data for 50,000 Individuals From the MyHeart Counts Study | Hershman et al, 2019 [8] | 40,017 | N/A^a^ | iPhone | No | 14 |
| The Asthma Mobile Health Study, a Large-Scale Clinical Observational Study Using ResearchKit | Chan et al, 2018 [5] | 6470 | 24 | iPhone | No | 16 |
| A Feasibility Study of the Burden of Disease of Atopic Dermatitis Using a Smartphone Research Application, myEczema | Shah et al, 2020 [25] | 519 | N/A | iPhone | No | 33 |
| The Effect of Digital Physical Activity Interventions on Daily Step Count: a Randomised Controlled Crossover Substudy of the MyHeart Counts Cardiovascular Health Study | Shcherbina et al, 2019 [26] | 1075 | N/A | iPhone | No | 18 |
| Clickotine, A Personalized Smartphone App for Smoking Cessation: Initial Evaluation | Iacoviello et al, 2017 [27] | 416 | 8 | iPhone | Yes | 88 |
| The Use and Effectiveness of Mobile Apps for Depression: Results From a Fully Remote Clinical Trial | Arean et al, 2016 [28] | 626 | 12 | iPhone and Android | No | 30 |
| A Smartphone App Designed to Help Cancer Patients Stop Smoking: Results From a Pilot Randomized Trial on Feasibility, Acceptability, and Effectiveness | Bricker et al, 2020 [29] | 59 | 8 | iPhone and Android | Yes | 92 |
| mHealth for Schizophrenia: Patient Engagement With a Mobile Phone Intervention Following Hospital Discharge | Ben-Zeev et al, 2016 [30] | 342 | 2 | Android | No | 92 |
| Characterizing User Engagement With Health App Data: a Data Mining Approach | Serrano et al, 2017 [31] | 1,011,008 | 288 | iPhone and Android | No | 8 |
| Clinically Meaningful Use of Mental Health Apps and its Effects on Depression: Mixed Methods Study | Zhang et al, 2019 [32] | 301 | 9 | Android | No | 96 |
| Guided Self-Help Works: Randomized Waitlist Controlled Trial of Pacifica, a Mobile App Integrating Cognitive Behavioral Therapy and Mindfulness for Stress, Anxiety, and Depression | Moberg et al, 2019 [33] | 500 | 4 | iPhone and Android | No | 20 |
| A Mobile Phone-Based Program to Promote Healthy Behaviors Among Adults With Prediabetes Who Declined Participation in Free Diabetes Prevention Programs: Mixed-Methods Pilot Randomized Controlled Trial | Griauzde et al, 2019 [34] | 69 | 12 | Not reported | Yes | 80 |
| Clinical Integration of a Smartphone App for Patients With Chronic Pain: Retrospective Analysis of Predictors of Benefits and Patient Engagement Between Clinic Visits | Ross et al, 2020 [35] | 253 | 10 | iPhone and Android | Yes | 72 |
| Mixed-Methods Analysis of Factors Impacting Use of a Postoperative mHealth App | Scott et al, 2017 [36] | 20 | 2 | iPhone and Android | Yes | 85 |
| Development and Evaluation of the See Me Smoke-Free Multi-Behavioral mHealth App For Women Smokers | Gordon et al, 2017 [37] | 151 | 12.9 | Android | Yes | 48 |
| Change in Glycemic Control With Use of a Digital Therapeutic in Adults With Type 2 Diabetes: Cohort Study | Berman et al, 2018 [38] | 118 | 12 | iPhone and Android | Yes | 89 |
| Usefulness of a Novel Mobile Diabetes Prevention Program Delivery Platform With Human Coaching: 65-Week Observational Follow-Up | Michaelides et al, 2018 [39] | 43 | 65 | Not reported | No | 80 |
| Mobile App Usage Patterns of Patients Prescribed a Smoking Cessation Medicine: Prospective Observational Study | Bruno et al, 2018 [40] | 1032 | 12 | iPhone and Android | No | 10 |
| Development and Implementation of a Person-Centered, Technology-Enhanced Care Model For Managing Chronic Conditions: Cohort Study | Petersen et al, 2019 [41] | 2894 | 40 | Not reported | No | 10 |
| Impact of a Mobile Health Application on User Engagement and Pregnancy Outcomes Among Wyoming Medicaid Members | Bush et al, 2017 [42] | 85 | 24 | Not reported | Yes | 100 |
| Efficacy of Osteoporosis Prevention Smartphone App | Ryan et al, 2020 [43] | 290 | 48 | iPhone | Yes | 90 |
| Get With the Program: Adherence to a Amartphone App for Smoking Cessation | Zeng et al, 2016 [44] | 416 | 1.1 | iPhone and Android | Yes | 85 |
| A Pilot Randomized Controlled Trial of a Web-Based Growth Mindset Intervention to Enhance the Effectiveness of a Smartphone App for Smoking Cessation | Sridharan et al, 2019 [45] | 398 | 8 | iPhone and Android | Yes | 92 |
| Trading Pounds for Points: Engagement and Weight Loss in a Mobile Health Intervention | Hales et al, 2017 [46] | 24 | 12 | Android | Yes | 62.5 |
| Feasibility of Smartphone-Based Education Modules and Ecological Momentary Assessment/Intervention in Pre-bariatric Surgery Patients | Mundi et al, 2015 [47] | 30 | 12 | iPhone and Android | No | 67 |
| Mobile App for Mental Health Monitoring and Clinical Outreach in Veterans: Mixed Methods Feasibility and Acceptability Study | Betthauser et al, 2020 [48] | 83 | 12 | Android | Yes | 42 |
| The Florida Mobile Health Adherence Project for People Living With HIV (FL-mAPP): Longitudinal Assessment of Feasibility, Acceptability, and Clinical Outcomes | Escobar-Viera et al, 2020 [49] | 37 | 4.3 | iPhone and Android | Yes | 72.5 |
| Early Weight Loss in a Standalone mHealth Intervention Predicting Treatment Success | Patel et al, 2019 [50] | 84 | 12 | iPhone and Android | No | 80 |
| OnTrack: Development and Feasibility of a Smartphone App Designed to Predict and Prevent Dietary Lapses | Forman et al, 2019 [51] | 43 | 8 | iPhone | Yes | 98 |
| A Group-Based Mobile Application to Increase Adherence in Exercise and Nutrition Programs: A Factorial Design Feasibility Study | Du et al, 2016 [52] | 58 | 8 | iPhone and Android | Yes | 72 |
| Rams Have Heart, a Mobile App Tracking Activity and Fruit and Vegetable Consumption to Support the Cardiovascular Health of College Students: Development and Usability Study | Krzyzanowski et al, 2020 [53] | 55 | 15 | iPhone and Android | Yes | 47 |
| Formative Evaluation of Participant Experience With Mobile eConsent in the App-Mediated Parkinson mPower Study: A Mixed Methods Study | Doerr et al, 2017 [6] | 9846 | 24 | iPhone | No | 28 |
| Feasibility of Obtaining Measures of Lifestyle From a Smartphone App: The MyHeart Counts Cardiovascular Health Study | McConnell et al, 2017 [7] | 20,345 | 1 | iPhone | No | 9 |
| Weight Loss Efficacy of a Novel Mobile Diabetes Prevention Program Delivery Platform with Human Coaching | Michaelides et al, 2016 [54] | 54 | 24 | Not reported | No | 84 |
| A Lower Leg Physical Activity Intervention for Individuals With Chronic Venous Leg Ulcers: Randomized Controlled Trial | Kelechi et al, 2020 [55] | 24 | 6 | Not reported | Yes | 33 |
| A Fully Automated Conversational Artificial Intelligence for Weight Loss: Longitudinal Observational Study Among Overweight and Obese Adults | Stein et al, 2017 [56] | 70 | 48 | iPhone and Android | No | 84 |
| Usability and Feasibility of a Smartphone App to Assess Human Behavioral Factors Associated with Tick Exposure (The Tick App): Quantitative and Qualitative Study | Fernandez et al, 2019 [57] | 1468 | 20 | iPhone and Android | No | 49 |
| A Mobile Just-in-Time Adaptive Intervention for Smoking Cessation: Pilot Randomized Controlled Trial | Hébert et al, 2020 [58] | 81 | 13 | Android | Yes | 96 |
| Characteristics and Usage Patterns Among 12,151 Paid Subscribers of the Calm Meditation App: Cross-Sectional Survey | Huberty et al, 2019 [20,59] | 12,151 | 4 | iPhone and Android | Yes | Not reported |
| Use of Mental Health Apps by Patients With Breast Cancer in the United States: Pilot Pre-Post Study | Chow et al, 2020 [60] | 40 | 7 | iPhone and Android | Yes | 58 |
| Mobile Delivery of the Diabetes Prevention Program in People With Prediabetes: Randomized Controlled Trial | Toro-Ramos et al, 2020 [61] | 202 | 20 | iPhone and Android | Yes | 82 |
| Utilizing Digital Health to Collect Electronic Patient-Reported Outcomes in Prostate Cancer: Single-Arm Pilot Trial | Tran et al, 2020 [62] | 29 | 12 | iPhone | No | 86 |
| Use of a Smartphone-Based Mobile App for Weight Management in Obese Minority Stroke Survivors: Pilot Randomized Controlled Trial With Open Blinded End Point | Ifejika et al, 2020 [63] | 36 | 12.9 | Not reported | No | 69 |
| A Mobile Phone-Based Health Coaching Intervention for Weight Loss and Blood Pressure Reduction in a National Payer Population: A Retrospective Study | Mao et al, 2017 [64] | 763 | 16 | iPhone and Android | No | 83 |
| A Clinically Integrated mHealth App and Practice Model for Collecting Patient-Reported Outcomes between Visits for Asthma Patients: Implementation and Feasibility | Rudin et al, 2019 [65] | 26 | 25 | iPhone and Android | Yes | 92 |
| Using Smartphones to Improve Treatment Retention Among Impoverished Substance-Using Appalachian Women: A Naturalistic Study | Johnston et al, 2019 [66] | 98 | 24 | Android | Yes | 47 |
| Using the Habit App for Weight Loss Problem Solving: Development and Feasibility Study | Pagoto et al, 2018 [67] | 27 | 8 | Android | Yes | 67 |
| A Novel Approach for Fully Automated, Personalized Health Coaching for Adults with Prediabetes: Pilot Clinical Trial | Everett et al, 2018 [68] | 55 | 12.9 | iPhone and Android | No | 86 |
| Multicomponent mHealth Intervention for Large, Sustained Change in Multiple Diet and Activity Risk Behaviors: The Make Better Choices 2 Randomized Controlled Trial | Spring et al, 2018 [69] | 212 | 12 | Not reported | Yes | 60 |
| The SMARTER Pilot Study: Testing Feasibility of Real-Time Feedback for Dietary Self-Monitoring | Burke et al, 2017 [70] | 39 | 12 | Android | Yes | 74 |
| Single-arm Trial of the Second Version of an Acceptance & Commitment Therapy Smartphone Application for Smoking Cessation | Bricker et al, 2017 [71] | 99 | 0.25 | iPhone and Android | Yes | 85 |
| Leveraging Positive Psychology to Support Smoking Cessation in Nondaily Smokers Using a Smartphone App: Feasibility and Acceptability Study | Hoeppner et al, 2019 [72] | 30 | 3 | Android | Yes | 97 |
| PositiveLinks: A Mobile Health Intervention for Retention in HIV Care and Clinical Outcomes with 12-Month Follow-Up | Dillingham et al, 2018 [73] | 77 | 48 | Android | Yes | 79 |

^a^N/A: not applicable.
